# Pyrolysis of fatty acids derived from hydrolysis of brown grease with biosolids

**DOI:** 10.1007/s11356-020-09041-3

**Published:** 2020-05-03

**Authors:** Mehdi Omidghane, Mattia Bartoli, Justice Asomaning, Lin Xia, Michael Chae, David C. Bressler

**Affiliations:** Department of Agricultural, Food and Nutritional Science, University of Alberta, Edmonton, T6G 2P5 Canada

**Keywords:** Thermal conversion, Microreactor, Pyrolysate, Sulphur, Renewable fuels, Drop-in fuels

## Abstract

The escalating generation of biosolids and increasing regulations regarding their safe handling and disposal have created a great environmental challenge. Recently, biosolids have been incorporated into the hydrolysis step of a two-step thermal lipid conversion process to act as water replacement in the production of renewable chemicals and fuels. Here, the hexane extract recovered from hydrolysis of biosolids, lipids from brown grease hydrolyzed using either water (control) or biosolids as a water replacement, was pyrolyzed at 410–450 °C for 2 h. The product distribution and composition were not significantly different when biosolids were used to hydrolyze brown grease instead of water. The liquid product consisted mainly of alkanes, alkenes, aromatics, and cyclic compounds similar to those in petroleum-derived liquid fuels. However, the use of biosolids as a water substitute resulted in a significant increase in sulphur content of the pyrolysate, which will necessitate processes to reduce the sulphur content before or after pyrolysis. Nevertheless, the pathways proposed in this paper are considered as potentially economically viable approaches to not only resolve the issues associated with disposal of biosolids but also to produce renewable hydrocarbons for fuel and chemical applications.

**Figure:**
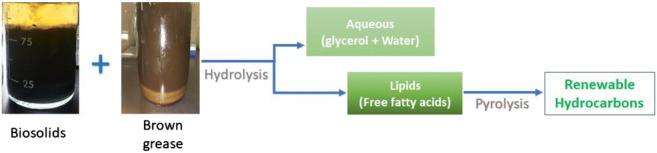
Graphical abstract

Graphical abstract

## Introduction

Biosolids are byproducts of all municipal wastewater treatment plants. Over the last decade, the generation of biosolids has increased continuously and substantially due to the rapid population growth in cities. Thus, the handling and safe disposal of biosolids have become a great challenge for communities around the world (Collivignarelli et al. 2019; Fytili and Zabaniotou 2008). Iglesias and Morales (2012) estimated that more than 20 million tonnes of dry biosolids are produced worldwide every year in 2011 and an estimated 13 million tonnes will be produced by 2020 in just the European Union (Kominko et al. 2017). The cost of biosolids processing can often be more than 50% of the total wastewater treatment costs (Collivignarelli et al. 2019; Rulkens 2007; Zhao et al. 2019). One of the most important issues for biosolids handling is that they contain pathogens and other microbial concerns, and thus should be sterilised prior to any further processing or release (Reinthaler et al. 2003). In this regard, various routes for biosolids handling have been evaluated including disposal after costly processing (Kelessidis and Stasinakis 2012; Zhao et al. 2019), or use in agriculture and land reclamation/restoration (Cantarero et al. 2017; Eid et al. 2019; Li et al. 2020; Lu et al. 2012; Yang et al. 2018). These applications are limited and associated with some drawbacks. For example, the use of biosolids in agricultural applications is restricted in many countries of the world due to the bad odour, as well as the release of heavy metals and toxic matter (Lu et al. 2012; Riaz et al.^2020^). In terms of heavy metals, biosolids often contain high levels of zinc, lead, copper, chromium, nickel, cadmium, and mercury (Hsiau and Lo 1998; Tarpani et al. 2020) that can be potentially harmful to the environment (McGrath et al. 1995; Riaz et al. 2020).

Other alternative applications have been previously explored, mostly in order to find an efficient way of extracting the energy and chemical content of biosolids (Rulkens 2007). These applications covered a wide range of thermal processes (Zhang et al. 2014) such as pyrolysis (Bridle and Pritchard 2004; Inguanzo et al. 2002), biodiesel production through lipid extraction and transesterification (Melero et al. 2015; Siddiquee and Rohani 2011), supercritical water oxidation (Svanström et al. 2004), and gasification (Arnold et al. 2017; Hamilton 2007; Jaeger and Mayer 2000; Trabelsi et al. 2017). The reason for testing these methods is that biosolids contain organic matter that can be potentially converted into chemicals or a renewable source of energy. A thermal process could also potentially result in the destruction of pathogens and easier heavy metal removal.

Pyrolysis is one of the most popular techniques among the thermo-chemical methods, as it is relatively simple and results in a liquid product (Jahirul et al. 2012; Radlein and Quignard 2013). In this process, organic matter in the feedstock is decomposed through a series of reactions occurring at a relatively high temperature and in the absence of oxygen. The pyrolysis of biosolids at various conditions has been investigated by several researchers (Fonts et al. 2012; Huang et al. 2014; Liu et al. 2018), but due to additional challenges regarding biosolids, research in this field is limited compared with pyrolysis of other forms of biomass. The most important problem is that the raw untreated biosolids contain large volumes of water, which should be removed prior to pyrolysis. However, the efficiency of the process in terms of energy recovery will be substantially decreased by this pretreatment step. In addition, the product is a highly oxygenated chemically unstable liquid that is not compatible with current fuels and requires further upgrading (Alvarez et al. 2016). Finally, the formed solids can end up in the final product causing reactor blockage (Pokorna et al. 2009). These issues will negatively impact the efficiency and economy of the process. In view of these drawbacks, there is a need to identify another cost-effective and sustainable technique that can convert raw untreated biosolids to liquid fuels and/or other value-added commodities.

An alternative pathway is the thermal processing of a mixture of biosolids and other types of feedstocks (Saw et al. 2012; Smoliński and Howaniec 2016). In particular, if biosolids can be used as a water source, then this would eliminate the need for biosolids dewatering. Asomaning et al. (2014c) suggested a two-step thermal process to convert lipid feedstocks such as beef tallow, yellow grease, and brown grease, into renewable chemicals and fuels. In this process, the feed was first hydrolyzed with water to convert esterified fatty acids to free fatty acids, which could then be pyrolyzed in the second step. The final product of such processes would be an organic liquid fraction consisting predominantly of *n*-alkanes. Data from our lab have shown that biosolids can be used as a water replacement during hydrolysis of brown grease to generate free fatty acids (Xia et al. 2019). It was shown that the use of biosolids as water a replacement during hydrolysis of brown grease resulted in statistically similar fatty acids conversion, recoveries, and composition when compared with the water control. However, it was also shown that some of the sulphur-containing compounds in the biosolids were carried over into the fatty acids resulting from hydrolysis. Furthermore, the conditions used for thermal hydrolysis of the biosolids/brown grease mixture can drastically improve settling rates of the solid material found in biosolids, which may support the development of wastewater treatment strategies to replace natural settling of biosolids in large lagoons (Chae et al. 2018; Xia et al. 2019).

The primary aim of this study is to investigate whether the lipid fraction obtained from hydrolysis of brown grease with biosolids can be used in pyrolysis reactions to produce renewable hydrocarbons, which can be used as fuels and chemicals. Pyrolysis conditions, such as temperature, were varied and their influence on the characteristics of the resulting products was studied.

## Materials and methods

### Materials

The feedstocks used for pyrolysis were fractions of the various organic phases recovered from hydrolysates as described by Xia et al. (2019) where the detailed characterises of the of the biosolids, brown grease, and the fatty acids resulting after hydrolysis are provided. Hydrolysis using biosolids was carried out in two situations: (1) using only raw untreated biosolids obtained from a wastewater treatment plant in Edmonton, AB, Canada; and (2) using a blend of biosolids and brown grease at a 1:1 mass ratio. We also used two other feedstocks as controls during pyrolysis: (1) the organic phase recovered from hydrolysis of brown grease with deionised water at a 1:1 mass ratio; and (2) oleic acid as a pure model fatty acid. Pentane (HPLC grade, > 99.9%), hexane (HPLC grade, > 99.9%), and the internal standard (methyl nonadecanoate; ≥ 98.0%) for gas chromatography (GC) of the liquid products were purchased from Sigma-Aldrich (St. Louis, MO, USA). Diazomethane for derivatization of fatty acids was prepared using a Diazald kit (Sigma-Aldrich, St. Louis, MO, USA) following the manufacturer’s procedures. Diazald (N-methyl-N-nitroso-*p*-toluenesulfonamide) used for diazomethane preparation was purchased from TLC Pharmaceutical Standards Ltd. (Aurora, ON, Canada). Air, N_2_, H_2_, and He gases were purchased from Praxair (Praxair Inc., Danbury, CT, USA).

### Pyrolysis

Pyrolysis reactions were conducted in 15-mL batch microreactors made from ¾ inch stainless steel Swagelok (Edmonton, Alberta, Canada) fittings and tubing. Approximately 1 g of material was loaded into a clean microreactor, which was then sealed, purged with nitrogen at 500 psi, and tested for leaks. The microreactor was then heated in a sand bath (Techne, Burlington, NJ, USA) at the desired temperature (410, 430, and 450 °C) for a period of 2 h. The microreactor was immediately submerged in a bucket of water at room temperature to terminate any further reactions. Following the cooling step, the microreactor was cleaned and dried using compressed air. Each experimental condition was replicated three times.

The microreactors were weighed before and after releasing the gas product to determine the mass of gas generated. After venting, the microreactors were opened and 10 mL of internal standard solvent solution (1.3 mg/mL methyl nonadecanoate in pentane) was added to each microreactor to dissolve and dilute the product with the exception of samples used in analysis of sulphur and phosphorus, where the liquid product was transferred to a glass vial without addition of the internal standard solution. The content was thoroughly mixed with a glass agitator. After mixing, the microreactor was capped and left at room temperature for 15 min to allow any solids to settle. The liquid content was then poured into sample vials and capped with a Teflon-lined screw cap and then stored at 4 °C prior to analysis. Any solid material left in the reactor was considered as pentane insoluble residues. To measure the amount of solid residue, the microreactor was left in a fume hood overnight until all the solvent had evaporated. The weight difference before and after cleaning was considered as the weight of solid residue.

### Product analysis

The solvent extracts from the pyrolysis products were analyzed using gas chromatography (GC) coupled to mass spectrometer for peak identification and flame ionization detector for peak quantification using an internal standard. The details of the sample preparations, instruments, supplies, and conditions are reported elsewhere (Omidghane et al. 2017).

### Analysis of sulphur and phosphorous in the liquid pyrolysis products

The liquid products isolated after pyrolysis of organic fractions obtained through hydrolysis of brown grease with water or biosolids were analyzed to determine the levels of sulphur and phosphorous present. For these samples, pyrolysis was performed as described in the “Pyrolysis” section at a temperature of 410 °C for 2 h. The samples were digested using microwave-assisted nitric acid digestion. Briefly, 5 mL of trace-metal grade HNO_3_ was added to a weighed sample in a PTFE digestion tube and digested in a MARS 5 microwave system (CEM, Matthews, NC, USA) for 24 h. Samples were diluted to a total volume of 25 mL and analyzed using a ThermoiCAP 6000 series inductively coupled plasma-optical emission spectrometer (ICP-OES; Thermo Fisher, Cambridge, UK) in the Analytical & Instrumentation Laboratory, Department of Chemistry, University of Alberta.

### Statistical analysis

Analysis of variance (ANOVA) with Tukey post hoc test was conducted with test sets at a 95% confidence level using Minitab 16 statistical software (Minitab Inc., State College, PA, USA).

## Results and discussion

In order to establish whether biosolids could be incorporated into lipid pyrolysis towards the generation of renewable hydrocarbon fuels and chemicals, a series of pyrolysis experiments were conducted. In the first set of experiments (“Pyrolysis of the organic phase extracted from hydrolyzed biosolids” section), pyrolysis of the organic phase extracted from biosolids hydrolysates was performed to gain insight into the effects of pyrolysis on organic materials derived exclusively from biosolids. For these experiments, a model fatty acid, oleic acid, was also pyrolyzed as a control. Oleic acid is a mono-unsaturated free fatty acid that is commonly found in large quantities in plant lipids and oils. In addition, oleic acid is the most abundant fatty acid in brown grease and the second most abundant fatty acid in the organic phase extracted from biosolids hydrolysate used in this study; hence, it was chosen as the model fatty acid. In the second set of experiments (“Pyrolysis of organic fractions obtained from hydrolysis of brown grease with biosolids” section), the organic phase isolated from hydrolysates of brown grease generated using water or biosolids was pyrolyzed to determine whether biosolids could serve as a water replacement for lipid pyrolysis without impacting the pyrolysis product. In both sets of experiments, pyrolysis was conducted at three temperatures (410, 430, and 450 °C) to assess the effect of temperature on the pyrolysis products.

### Pyrolysis of the organic phase extracted from hydrolyzed biosolids

The organic phase that was acquired from hydrolysis of biosolids alone contains a small amount of free fatty acids (Xia et al. 2019). In this study, we subjected the organic phase from hydrolyzed biosolids to pyrolysis reactions at 410, 430, and 450 °C and then analyzed the products to determine how temperature impacts conversion of the organic material extracted from biosolids to hydrocarbon-based fuels. Previous experiments conducted on pyrolysis of model fatty acids such as oleic acid and stearic acid have shown that at high temperatures, the fatty acids undergo deoxygenation and other thermochemical reactions including cracking and isomerization, as well as aromatization, to form hydrocarbons (Asomaning et al. 2014a, b; Jenab et al. 2014; Maher et al. 2008). However, since biosolids are complex mixtures, other components may potentially impact pyrolysis, perhaps by acting as a catalyst to enhance desirable reactions, or conversely, by inhibiting hydrocarbons production.

#### Product distribution yields

**Fig. 1:**
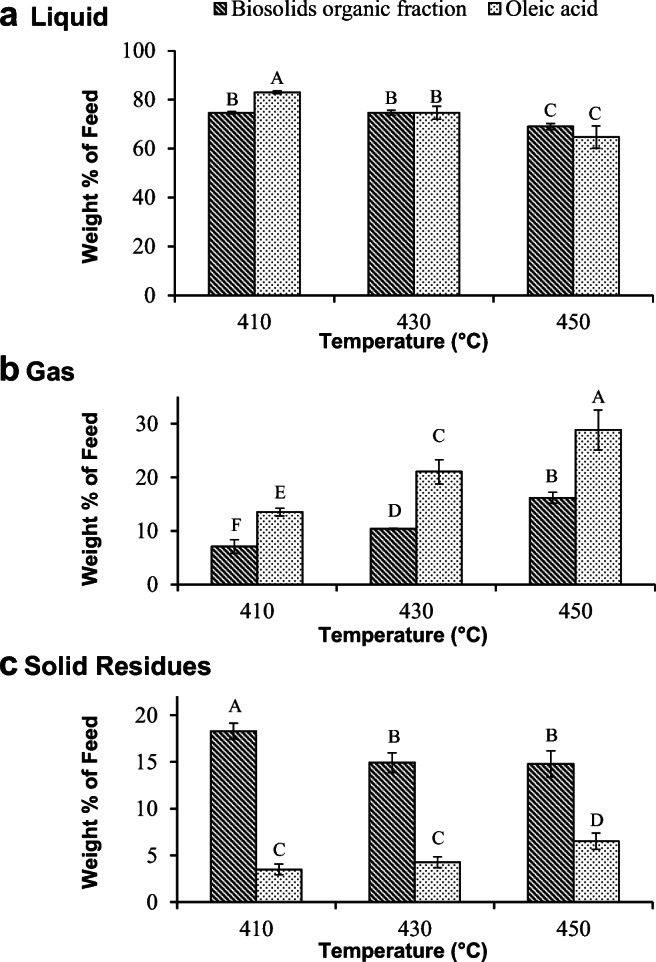
The yield of liquid (A), gas (B), and solid residues (C) produced from pyrolysis of the organic phase extracted from the biosolids hydrolysate or oleic acid. The bars represent the average of three replicates for each treatment ± standard deviation. For each of the three products, data with different letters are statistically different at a 95% confidence level

The pyrolysis reaction resulted in three products: liquid, gas, and solid residues. Among these three fractions, the liquid is the most important and valuable one for renewable fuel applications. The product yields were calculated as a percentage of the mass of pyrolysis feed. Figure 1 shows the liquid, gas, and solid yields obtained from pyrolysis of the organic phase from biosolids hydrolysates and oleic acid at 410, 430, and 450 °C. As seen in Fig. 1A, at 410 °C, the liquid yield of the product obtained from pyrolysis of the organic phase from biosolids hydrolysates was slightly lower than that obtained from pyrolysis of oleic acid. However, at 430 and 450 °C, the liquid yields were not significantly different between the two samples at a given temperature. Regarding the pyrolysis of oleic acid, the liquid yield was found to be decreasing with increasing temperature. In contrast, in the pyrolysis of the organic phase from biosolids hydrolysates, the liquid yield did not change with a temperature increase from 410 to 430 °C but decreased when the temperature was further increased to 450 °C.

Figure 1B shows the gas yield at different temperatures. The amount of gas produced increased as the temperature increased for both samples. This phenomenon was due to the increase in deoxygenation, which results in the production of CO and CO_2_, and cracking reactions, which are responsible for the production of light hydrocarbon gases such as methane, with rising temperatures. Figure 1C shows the solid residues that were collected in pyrolysis of oleic acid and the organic phase from hydrolyzed biosolids at different temperatures. At all temperatures, the solid residues produced from pyrolysis of the organic fraction from biosolids hydrolysates were significantly higher than those obtained from pyrolysis of oleic acid. The high amounts of residue could likely be attributed to the presence of substances that were not soluble in pentane. These substances are generally high molecular weight aromatic compounds that are formed as a result of polymerization of monoaromatic compounds (Asomaning et al. 2014a; Jenab et al. 2014; Maher and Bressler 2007). Xia et al. (2019) showed that the organic fraction from hydrolysis of biosolids contained a significant amount of oxygen-free hydrocarbons including aromatic compounds in addition to the free fatty acids. Thus, the presence of the aromatic compounds in the organic fraction from hydrolysis of biosolids could accelerate the formation of polyaromatic hydrocarbons and hence the amount of solids. In addition, Xia et al. (2019) reported the presence of compounds with molecular weights comparable with diacylglycerols. These would be expected to further impact the amount of solids formed during the pyrolysis of the organic fraction from biosolids hydrolysis through dimerization, oligomerization, and polymerization. Furthermore, the chromatogram derived from the liquid product generated through pyrolysis of the organic fraction obtained from hydrolysis of biosolids demonstrates the presence of substances with high molecular weight (retention time higher than 20 min) whereas in those obtained using oleic acid, there is the absence of any appreciable amounts of compounds besides the C18:0 peak.

When the temperature was increased to 450 °C, the solid residues from the organic fraction of hydrolyzed biosolids did not significantly change, whereas the solid residues from pyrolysis of oleic acid increased. This increase in solid residues from pyrolysis of oleic acid at 450 °C is in agreement with previously reported non-catalytic oleic acid pyrolysis, which is often attributed to the polymerization of aromatic compounds at this temperature (Asomaning et al. 2014a; Jenab et al. 2014; Maher and Bressler 2007); these reactions may cause an increase in residues.

#### Liquid fraction composition

**Fig. 2:**
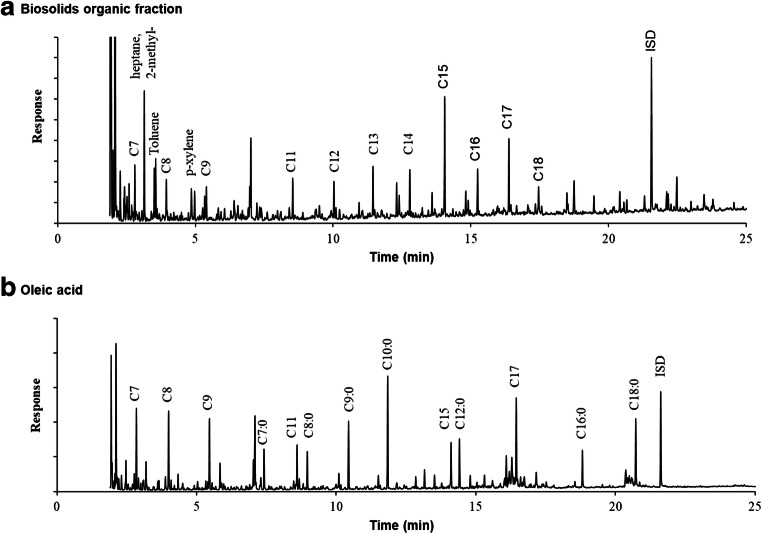
Identification of peaks in GC-MS chromatograms derived from the liquid product obtained following pyrolysis at 410 °C using the organic phase extracted from hydrolyzed biosolids (A) or oleic acid, a model fatty acid (B)

To evaluate the pyrolysis of the organic phase of hydrolyzed biosolids, the GC-MS chromatogram of the liquid product obtained through pyrolysis at 410 °C was compared with that obtained from pyrolysis of oleic acid under the same condition (Fig. 2). As seen in this figure, alkanes ranging from C7 to C18 hydrocarbons were detected in the liquid products. A similar observation was also made in the liquid pyrolysis products at 430 and 450 °C. It is important to note that the compounds reported here have molecular weights that are higher than *n*-hexane because the peaks for equal or smaller molecular weight compounds could not be analyzed due to solvent peak overlap. The product distribution of oleic acid pyrolysis obtained in this study was in agreement with that reported by Asomaning et al. (2014a) and showed that the main products were *n*-alkanes that are likely compatible with existing petroleum-derived fuels.

A quick comparison between the two chromatograms in Fig. 2 revealed that a similar profile of *n*-alkanes was generated for both samples. However, they differed in the amounts of other compounds such as fatty acids. In fact, it appeared that the liquid product obtained from pyrolysis of the organic phase of biosolids hydrolysates had a smaller number of fatty acids identified. A quantitative analysis will be presented below, but it is worth mentioning that the presence of fatty acids in the pyrolysis product is undesirable for its application in transportation fuels. Fatty acids increase the viscosity of fuel and cause increased engine deposits (Knothe 2009). Furthermore, fatty acids are difficult to remove during distillation as some fatty acid boiling points are similar to the boiling points of desirable hydrocarbons necessitating additional removal steps. Therefore, it is of great importance that the product would be ideally free of any fatty acids or at least contain a minimal amount. In addition to fatty acids, the product seemed to differ with regard to the quantities of the aromatics toluene and xylene formed. The pyrolysis of the organic phase extracted from hydrolyzed biosolids resulted in significantly greater amounts of total aromatics as compared with that obtained from oleic acid pyrolysis.

**Fig. 3:**
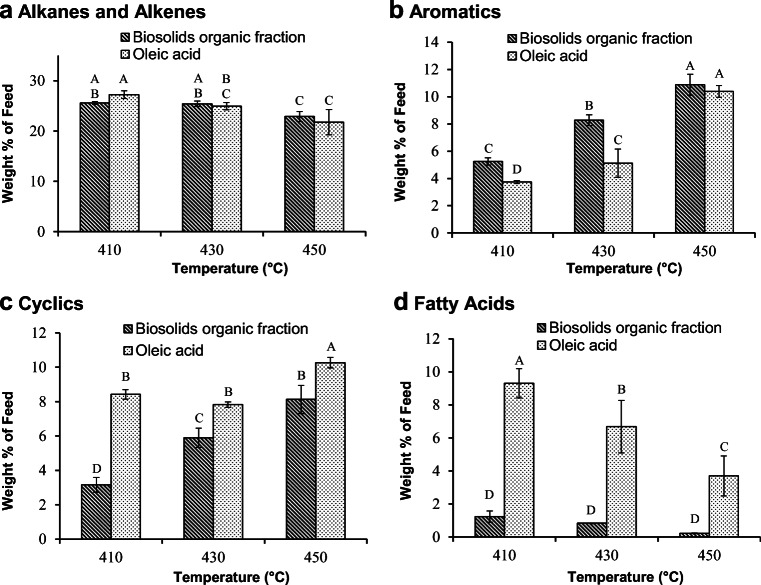
Mass percentages of C7-C22 alkanes and alkenes (A), aromatics (B), cyclic compounds (C), and total fatty acids (D) in the liquid fraction obtained through pyrolysis (at 410, 430, and 450 °C) of the organic phase extracted from biosolids hydrolysates or oleic acid. The bars are the average of three replicates for each treatment ± standard deviation. For each of the products, data with different letters are statistically different at a 95% confidence level

The chemical composition of the liquid product provides insights into its properties and suitability for use as a feedstock for the production of transportation fuels. Thus, quantification of the components of the liquid fraction was also performed via GC-FID. The identified compounds found in the liquid fraction following pyrolysis of the organic phase of biosolids hydrolysates and oleic acid have been classified into four categories: (1) C7-C22 alkanes and alkenes (linear and branched); (2) cyclic hydrocarbons; (3) aromatic compounds; and (4) fatty acids. Quantification of these groups in the liquid product following pyrolysis at 410, 430, and 450 °C is shown in Fig. 3 for both oleic acid and the organic phase of biosolids hydrolysates.

**Fig. 4:**
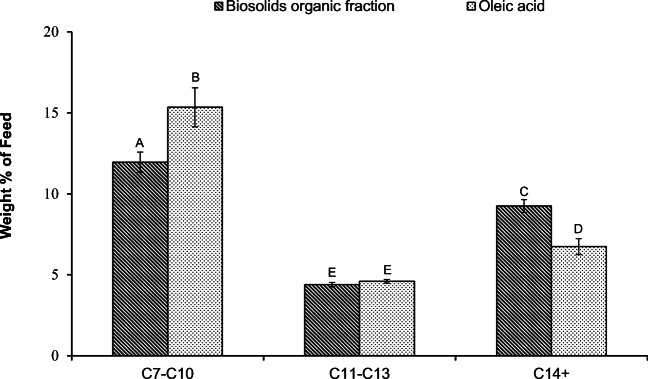
The distribution of alkanes and alkenes in the liquid fraction resulting from pyrolysis of the organic phase extracted from biosolids hydrolysates or oleic acid at 410 °C. The bars are the average of three replicates for each treatment ± standard deviation. Data with different letters are statistically different at a 95% confidence level

Data from Fig. 3 show that at all temperatures, the main products were alkanes and alkenes. The weight percent of this fraction in the product from the organic phase of biosolids hydrolysates was similar to that obtained from oleic acid at all temperatures. Alkanes and alkenes are considered as favourable constituents of the liquid product. The distribution of these compounds, however, determines the type of the fuel that can be produced from the feedstock upon distillation. Figure 4 shows the distribution of compounds in the alkanes and alkenes category in the liquid product obtained from pyrolysis of oleic acid and the organic phase of biosolids hydrolysates at 410 °C. All alkanes and alkenes detected in the liquid product were placed in three groups: (1) Light hydrocarbons ranging from C7 to C10 alkanes and alkenes, (2) middle range hydrocarbons from C11 to C13, and (3) the heavy molecules equal or larger than C14 alkanes and alkenes. For both feedstocks, the light hydrocarbons (C7–C10) were most abundant in the liquid product; however, the light hydrocarbons from the organic phase of biosolids hydrolysates were slightly lower than that observed for oleic acid (11.96 ± 0.62 vs. 15.34 ± 1.21 wt% of feed). Conversely, larger amounts of C14+ hydrocarbons were present in the liquid product from pyrolysis of the organic phase of biosolids hydrolysates (9.24 ± 0.39 vs. 6.74 ± 0.50 wt% of feed). The amounts of C11–C13 hydrocarbons were not significantly different (*p* > 0.05) between the organic phase of biosolids hydrolysate and oleic acid, accounting for 4.39 ± 0.14 wt% of feed and 4.60 ± 0.11 wt% of feed, respectively.

Figure 3 also shows that at 410 °C and 430 °C, the amount of aromatics was higher in the liquid product from the pyrolysis of the organic phase of biosolids hydrolysates compared with that from oleic acid; however, the levels were statistically similar at 450 °C. This suggests that the rate of aromatization reactions during pyrolysis of the organic phase of biosolids hydrolysates was higher than that of oleic acid at 410 and 430 °C, but the difference was negated at a higher temperature of 450 °C. However, the organic fraction from the hydrolysis of biosolids contained a reasonable amount of aromatic compounds (Xia et al. 2019), which may have influenced aromatization reactions during pyrolysis. The liquid product contained cyclics that were predominately 5-carbon and 6-carbon ring structured compounds. At all temperatures, the liquid product acquired from pyrolysis of the organic phase of biosolids hydrolysates contained significantly less cyclic compounds compared with those in the product from oleic acid. This is likely because the cyclization reactions were enhanced by the presence of double bonds in oleic acid (Asomaning et al. 2014b), while the organic fraction obtained from hydrolysis of biosolids contained a large amount of saturated fatty acids.

It is important to note that the residual fatty acid content of the liquid product resulting from pyrolysis of the organic phase of biosolids hydrolysates was found to be much lower than that observed for oleic acid at all three temperatures. The difference was greatest at 410 °C, where the product obtained from processing of the organic phase of biosolids hydrolysates contained 1.23 ± 0.34 wt% of feed, while the product from oleic acid was characterised with 9.31 ± 0.89 wt% of feed total fatty acids. Although the simplest explanation for the lower amount of fatty acids observed is a higher rate of conversion during the pyrolysis of the organic phase of biosolids hydrolysates, this is not likely the case here as a higher conversion rate should result in larger amounts of alkanes and alkenes (Omidghane et al. 2017), which was not observed. The organic fraction obtained from the hydrolysis of biosolids was shown to contain a relatively low amount of oxygen and fatty acid (Xia et al. 2019). Thus, the lower fatty acids content observed in the pyrolysis of the organic fraction from biosolids is the results of the low fatty acids content of the starting feed for the pyrolysis reaction.

### Pyrolysis of organic fractions obtained from hydrolysis of brown grease with biosolids

The pyrolysis of the organic fractions from the hydrolysis of biosolids and oleic acid as a model fatty acid provided valuable insight into the pyrolysis reaction. However, due to the extremely low amounts of organic compounds present in the biosolids, the direct pyrolysis of just the organic fraction obtained from hydrolysis of biosolids would be cost prohibitive (Xia et al. 2019). Thus, the use of biosolids as water replacement in the two-step thermal conversion of lipids to renewable fuels and chemicals will be a more economical approach. Xia et al. (2019) successfully demonstrated that biosolids could be used as a water substitute for the hydrolysis of brown grease to free fatty acids without affecting the degree of hydrolysis. The lipid fractions resulting from the hydrolysis of brown grease with biosolids or water (control) were isolated and subsequently subjected to pyrolysis at 410, 430, or 450 °C. A detailed analysis of the pyrolysis products is described in the “Product distribution yields” and “Liquid fraction composition” sections.

#### Product distribution yields

**Fig. 5:**
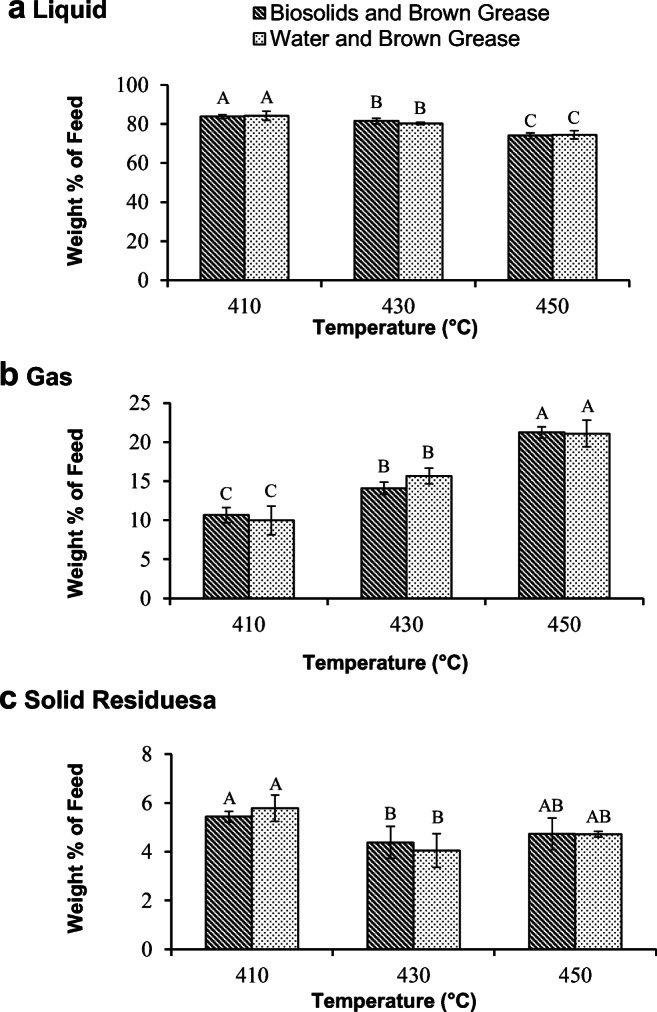
The yield of liquid (A), gas (B), and solid residues (C) acquired from pyrolysis of organic matter obtained from hydrolysis of brown grease with biosolids or water. Pyrolysis was performed at three different temperatures: 410, 430, and 450 °C. The bars are the average of three replicates for each treatment ± standard deviation. For each of the three products, data with different letters are statistically different at a 95% confidence level

The pyrolysis reaction resulted in three products: liquid, gas, and solid residues. Figure 5, which shows the yield of these products following pyrolysis, indicates that the liquid yield decreased with temperature for both pyrolysis feeds, while the amount of gaseous products increased. This was expected as the severity of cracking reactions increases with increasing temperature, resulting in a higher amount of gaseous and a lower amount of liquid products.

The most significant finding from these experiments is that at all three temperatures tested, the amount of all products (gas, liquid, and solid residues) obtained from pyrolysis of the organic phase derived from hydrolysis of brown grease with biosolids were statistically similar to those acquired from organic matter obtained from brown grease hydrolyzed with water. This further supports the notion that the water used for hydrolysis of brown grease could be replaced with biosolids as the yields observed in the two systems were the same.

#### Liquid fraction composition

**Fig. 6:**
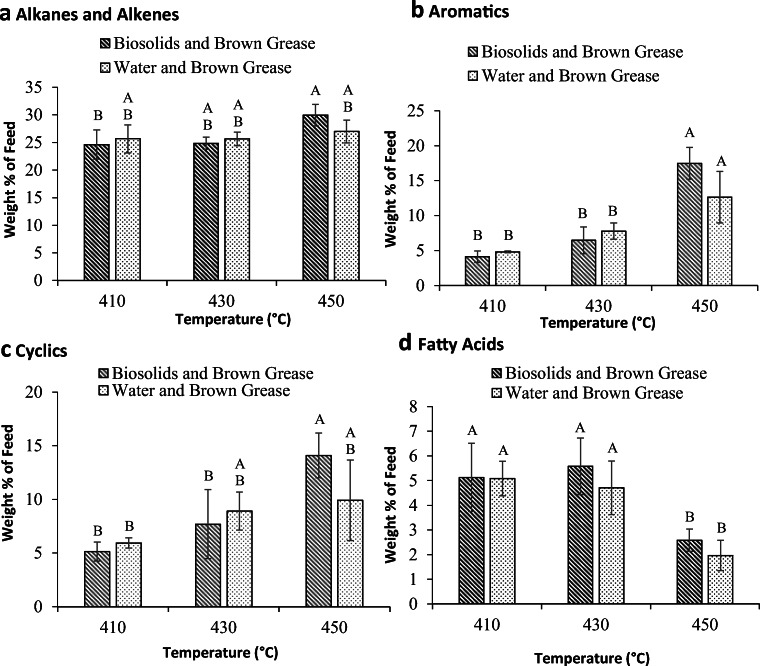
Mass percentages of C7–C17 alkanes and alkenes (A), aromatics (B), cyclic compounds (C), and total fatty acids (D) in the liquid fraction obtained from pyrolysis (at 410, 430, and 450 °C) of the organic phases that were acquired from hydrolysis of brown grease with biosolids or water. The bars are the average of three replicates for each treatment ± standard deviation. For each of the products, data with different letters are statistically different at a 95% confidence level

It is important to look into the composition of the liquid product to examine the impact of incorporating biosolids into the hydrolysis step of lipid pyrolysis on the quality of the product. Biosolids contain some complex compounds that could potentially be extracted along with the organic matter. These substances may negatively impact the pyrolysis reactions. Figure 6 shows the weight percent of the different classes of compounds (alkanes and alkenes, aromatics, cyclics, and fatty acids) in the liquid fraction obtained from pyrolysis of samples at 410, 430, and 450 °C. Similar to the pyrolysis of oleic acid and the organic fraction from biosolids hydrolysate (Fig. 3), the alkanes and alkenes were the major compounds in the liquid product from pyrolysis of samples obtained from hydrolysis of brown grease with water and biosolids. The *n*-alkanes with carbon numbers between 7 and 18 were the predominant of this class of compounds accounting for 16–18% of the liquid fraction of the water hydrolyzed brown grease and 15 to 21% of the biosolids hydrolyzed brown grease. No significant difference was observed (*p* ≥ 0.05) between the water hydrolyzed and biosolids hydrolyzed brown grease at a given temperature.

The fatty acid content of the liquid product was not significantly different between brown grease hydrolyzed with either water or biosolids (Fig. 6). However, at 450 °C, there were significantly less acids when compared with 410 °C and 430 °C. This observation was expected as a higher temperature of reaction has been shown to promote deoxygenation (Asomaning et al. 2014b) resulting in lower residual fatty acids in the liquid product. The fatty acids ranged from C4 to C18 and with the exception of C18, which had some unsaturated fatty acids (C18:1), all the acids were saturated. Thus, based on the fatty acid composition of both the water and biosolids hydrolyzed brown grease, some of the fatty acids (C4 to C11) were the product of the pyrolysis reaction. At 410 °C and 430 °C, C12 and higher acids accounted for more than 60% of the acids while at 450 °C, C12 and higher acids accounted for about 30% of the acids fraction. This observation could again be explained in terms of the severity of the cracking reactions at the higher temperature resulting in lower molecular weight compounds as previous reported (Asomaning et al. 2014a, b).

The cyclic compounds were composed of 3-carbon ring structures to 14-carbon ring structures. However, the 5-carbon and 6-carbon were the predominant structures accounting for 65 to 85% of the cyclic compounds, with the percentage increasing with temperature. It is worth noting that the cyclic compounds identified under all conditions were substituted similar to previously reported studies (Asomaning et al. 2014b). Similar to the other classes of compounds, no significant difference was observed between water hydrolyzed and biosolids hydrolyzed brown grease at a given temperature.

Aromatic compounds were also detected in the liquid product. The amounts of aromatics did not change significantly by increasing the temperature from 410 to 430 °C, but increased when the temperature was raised to 450 °C. The aromatic compounds were more or less evenly distributed between monoaromatic compounds containing one aromatic ring structure and polyaromatic compounds at 410 °C, whereas at 430 °C and 450 °C, monoaromatic compounds predominated accounting for up to 78% of the aromatic compounds.

Based on the results from this study, at all temperatures, a significant difference was not observed between the liquid product composition of the two systems, again suggesting that the hydrolysis of brown grease with biosolids instead of water did not impact the product obtained through subsequent pyrolysis of the organic fraction. This further suggests that biosolids can be used to as water replacement for hydrolysis of lipids in the two-step thermal lipid conversion technology for producing renewable fuels and chemicals.

#### Quantification of sulphur and phosphorous in the liquid pyrolysis product

Biosolids are known to contain phospholipids and sulpholipids (Chae and Tabatabai 1981; Kovar and Grant 2011), which can have a negative impact on the quality of the liquid fuel product obtained through lipid pyrolysis by increasing levels of phosphorous and sulphur, respectively. In the European Union and the USA, the amount of sulphur in gasoline and diesel must be below 10 ppm (Iruretagoyena and Montesano 2018); such low limits minimise production of sulphur oxides (SO_*x*_) that can lead to acid rain. Alternatively, the presence of phosphorous in fuel can lead to increased particulate emissions, which can damage engines (Mittelbach 1996). Xia et al. (2019) determined that the organic fraction extracted from biosolids hydrolysates contained 1.9 ± 0.6 wt% of sulphur and 397 ± 9 ppm of phosphorous. To determine how fuel quality is impacted by using biosolids as a water replacement for hydrolysis of lipids during fatty acids pyrolysis, we examined the levels of sulphur and phosphorus in the liquid product obtained after pyrolysis of the lipids fractions derived from the hydrolysis of brown grease with water or biosolids.

The liquid product from the pyrolysis (410 °C for 2 h) of the organic fraction obtained from hydrolysis of brown grease with water showed a sulphur concentration of 63.4 ± 9.7 ppm and a phosphorous content of 29.3 ± 7.6 ppm. When biosolids was used in place of water, the sulphur and phosphorous concentrations rose to 239 ± 21 ppm and 771.6 ± 83 ppm, respectively. Since the levels of sulphur in this liquid product are much higher than allowable for transportation fuels, the liquid pyrolysis product, or the organic fraction used as feedstock for pyrolysis, will likely need to be subjected to desulphurization approaches. This may include hydrodesulphurization, which is a method typically employed by petrochemical refineries, as well as novel approaches such as biodesulphurization, adsorption, extractive and oxidative desulphurization, and the use of ionic liquids (Ibrahim et al. 2017). Future research will focus on developing and optimizing desulphurization strategies to reduce sulphur in the product and/or feed prior to pyrolysis.

The high levels of phosphorous present in the liquid pyrolysis product when biosolids were used as a water replacement for hydrolysis of brown grease would also have a negative effect on fuel quality. The increase in phosphorous levels may stem from the formation of light organophosphates according to mechanism proposed by (Zegers and Fisher 1998). In this case, the formation of organophosphates could be ascribed to the higher starting phosphorous concentration, which would promote formation of high amounts of phosphonyl radicals that would lead to the incorporation of phosphorous into organic matter.

## Conclusions

When biosolids were used instead of water during the hydrolysis of brown grease and the resulting organic phase was subjected to pyrolysis, there were no differences in terms of product distribution (i.e. liquid, gas, and solid residues) and composition of the liquid product compared with when water was used in the hydrolysis step. Furthermore, both systems had similar responses to increases in temperature, namely a decrease in liquid product and an increase in gaseous product, with a decrease in residual fatty acids. While the use of biosolids resulted in an increase of sulphur and phosphorous content in the liquid pyrolysis product, incorporation of available techniques for desulphurization (i.e. hydrodesulphurization) and phosphorous removal could improve the properties of the liquid pyrolysis product for fuel applications. Thus, this research demonstrates the feasibility of incorporating biosolids into lipid pyrolysis for production of renewable hydrocarbons.
